# Effect of hydroxyapatite nanoparticles on enamel remineralization and estimation of fissure sealant bond strength to remineralized tooth surfaces: an in vitro study

**DOI:** 10.1186/s12903-019-0785-6

**Published:** 2019-05-28

**Authors:** Mahtab Memarpour, Fereshteh Shafiei, Azade Rafiee, Mina Soltani, Mohammad Hossein Dashti

**Affiliations:** 1 Oral and Dental Disease Research Center, Department of Pediatric Dentistry, School of Dentistry, Shiraz University of Medical Sciences, Shiraz, Iran; 2 Oral and Dental Disease Research Center, Department of Operative Dentistry, School of Dentistry, Shiraz University of Medical Sciences, Shiraz, Iran; 3 Department of Restorative Sciences and Biomaterials, Henry M. Goldman School of Dental Medicine, Boston University, Boston, MA USA

**Keywords:** Nano-hydroxyapatite, Pit and fissure sealant, Energy-dispersive X-ray spectroscopy, Remineralization

## Abstract

**Background:**

The management of noncavitated caries lesions before sealant therapy is a clinical challenge when the tooth needs sealant application. Sealing noncavitated carious lesions in pits and fissures may lead to failure of the fissure sealant (FS) due to incomplete sealing. Therefore the use of remineralizing agents such as nanoparticles has been suggested. This study investigated the ability of hydroxyapatite nanoparticles (nano-HA) to remineralize enamel, and their effect on sealant microleakage and shear bond strength (SBS).

**Methods:**

A total of 192 third molars were demineralized and pretreated with two concentrations of nano-HA with and without sodium hexametaphosphate (SHMP), followed by phosphoric acid etching and resin FS application. The study groups were 1) etching + FS, 2) etching + nano-HA 0.15% + FS, 3) etching + nano-HA 0.03% + FS, 4) etching + mixture of nano-HA 0.15% and SHMP 0.05% + FS, 5) etching + mixture of nano-HA 0.03% + SHMP 0.01% + FS. The laboratory tests included microleakage in 50 teeth, scanning electron microscopy (SEM) evaluation in 10 samples, and SBS in 100 samples. Enamel remineralization changes were evaluated in 32 teeth with energy-dispersive X-ray spectroscopy (EDS) and field emission scanning electron microscope (FESEM).

**Results:**

Nano-HA enhanced the SBS to remineralized enamel in a large percentage of nanoparticles. Mean SBS in group 2 was significantly greater than in groups 1, 3 and 4 (all *P* < 0.05). SBS was related to nano-HA concentration: nano-HA 0.15% yielded greater SBS (16.8 ± 2.7) than the 0.03% concentration (14.2 ± 2.1). However, its effect on microleakage was not significant. Nano-HA with or without SHMP led to enhanced enamel remineralization; however, the Calcium (Ca)/Phosphate (P) weight percent values did not differ significantly between the groups (*P* > 0.05). SEM images showed that SHMP did not affect sealant penetration into the deeper parts of fissures. FESEM images showed that adding SHMP led to increased nanoparticle dispersal on the tooth surface and less cluster formation.

**Conclusions:**

The ultraconservative approach (combining nano-HA 0.15% and SHMP) and FS may be considered a minimal intervention in dentistry to seal demineralized enamel pits and fissures.

## Background

Pit and fissure sealing is an accepted method to prevent dental caries or arrest the progression of noncavitated carious lesions in the deep parts of occlusal surfaces. The number of bacteria and their production are reduced after pits and fissures are sealed [1]. However, some studies have recommended using a pit and fissure sealant on teeth with noncavitated lesions [2, 3]. In some cases, practitioners may be uncertain about sealing noncavitated carious lesions or incipient caries such as lesions in the deepest parts of pits and fissures. In addition, some dentists have concerns about applying the sealant on decalcified enamel in the deep parts of occlusal grooves or lateral walls of fissures, which are not detectable by visual examination [4, 5]. Several methods are available for enamel pretreatment prior to sealant therapy, e.g. preparation with burrs, air abrasion or laser [6, 7]. Some researchers have reported the use of the resin infiltration technique as a microinvasive approach to preserve demineralized enamel. A low-viscous resin infiltrant combined with a flowable composite resin has been used to seal the porous occlusal subsurface in initial caries lesions. This technique increased marginal adaption and internal integrity compared to the use of a conventional flowable composite as a fissure sealant [8].

Other methods use the benefits of remineralizing agents such as pit and fissure sealants containing fluoride, amorphous calcium phosphate or nanoparticles [2, 9–16]. With the introduction of nanotechnology, some researchers have tested the use of nanoparticles in restorative and preventive dentistry [17–19]. One type of nanoparticle used in dentistry is nanohydroxyapatite (nano-HA). Nano-HA was considered promising because of its similarity to the bone and mineral structure of teeth, biocompatibility, and bioactivity [19, 20]. The particles are “similar in morphology and crystal structure to dental apatite” [21]. The remineralization characteristics of nano-HA particles have been reported in studies in which nanoparticles were added to a glass ionomer or other restorative materials [20, 22]. Toothpastes containing nano-HA showed greater remineralization effects in dentin compared to amine fluoride toothpastes [23].

To improve nano-HA infiltration and deagglomerate nano-HA particles, the addition of the deflocculant agent sodium hexametaphosphate (SHMP) to nano-HA has been recommended [24]. This results in more efficient hydroxyapatite crystal remineralization by providing a smaller particle size. Sodium hexametaphosphate is widely used as a food additive (E number E452i), water softener, and dispersing agent to break down clay and other soil types [25]. In the present study the effects of nano-HA with and without SHMP were compared.

Some studies have investigated commercial fissure sealants with nanoparticles added to sealant materials. These studies focused on the mechanical and chemical properties of the sealants as well as their antibacterial and remineralization characteristics [13–16]. However, no data are available on the use of nano-HA with pit and fissure sealants in demineralized enamel. The aim of the present study was to investigate the efficacy of nano-HA applied prior to the pit and fissure sealant to remineralize artificial enamel caries lesions. Our hypothesis (H0) was that two concentrations of nano-HA would be similar to conventional fissure sealant in their ability to infiltrate demineralized enamel without influencing the sealant’s sealing ability or bond strength of the sealant to tooth surface. The null hypothesis was tested against an alternative hypothesis (HA) that differences would be found. We compared microleakage and shear bond strength (SBS) after treatment with nano-HA. In addition, we studied the changes in enamel mineral composition and nano-HA particle microstructure with energy dispersive X-ray spectroscopy (EDS) and field emission scanning electron microscopy (FESEM). Scanning electron microscopy (SEM) was used to observe the sealant–enamel interface.

## Methods

The research protocol was approved by the Human Ethics Review Committee of the School of Dentistry, Shiraz University of Medical Sciences. A total of 192 sound extracted third molars were cleaned and disinfected by immersion in 0.1% chloramine T solution for 4 weeks. The aim of the study was explained to patients who decided to have their third molar extracted, and their informed consent in writing was obtained. The teeth were observed under a stereomicroscope (Motic K, Wetzlar, Germany) at 20× to rule out those with defects, cracks or caries.

### Early caries lesions

Briefly, for microleakage and SEM assessment, each tooth was randomly selected and demineralized by immersion in 15 mL demineralization solution at 37 °C for 96 h. For SBS, EDS and FESEM studies, the specimens were demineralized followed by polishing and sonication of the buccal and lingual enamel surfaces. The demineralization solution contained 6 μM methyl hydroxydiphosphonate, 50 mM lactic acid solution, 3 mM calcium chloride dihydrate and 3 mM potassium dihydrogen phosphate, and the final pH was adjusted to 4.5 [26]. The solution was replaced with freshly made solution after 48 h. After 96 h, each sample was washed with deionized water for 20 s, air dried and subjected to different conditions as detailed below.

### Preparation of the nano-HA solution

Before starting tooth treatments, nano-HA particle size (nano-HA, Merck, nGimat, Darmstadt, Germany) was measured with a nanoparticle size analyzer (Horiba Ltd., Kyoto, Japan). The average diameter of the particles was recorded as 10.67 nm. Then two types of nano-HA were prepared, one with and one without SHMP (Merck, nGimat, Darmstadt, Germany).

The solution contained distilled water and ≥ 99.5% acetone (Acetone, Merck, nGimat, Darmstadt, Germany) as a solvent at a 1:1 ratio. The nano-HA and SHMP powder was measured and mixed at a 3:1 ratio and added to the solution. The prepared solutions contained nano-HA 0.15% or 0.03% (w/v) and SHMP at 0.05% or 0.01% (w/v). The amounts of powder and solvent needed to produce suitable concentrations of nano-HA and SHMP for testing were determined according to previous research [24] and a pilot study carried out in our laboratory.

### Experimental groups

After demineralization, the enamel was pretreated as follows and fissure sealant was applied:

Group 1 (control): The enamel was etched with phosphoric acid 35% (3 M, ESPE, St. Paul, MN, USA) for 20 s, rinsed and dried under a weak air stream. Then an unfilled fissure sealant (FS) (Clinpro, 3 M ESPE, St. Paul, Minn, USA) was applied and cured with a halogen light curing unit (Coltolux, Coltene, Whaledent, Altstaetten, Switzerland) at a power density of 550 mW/cm^2^ for 40 s.

Group 2 (nano-HA 0.15%): After the enamel was etched as described above for group 1, each sample was immersed in 5 mL of a solution that contained 0.15% nano-HA in a closed glass vial with continuous slow speed rotation (4 rmp) for 5 min to ensure that the nanoparticles remained in suspension and to avoid precipitation [24]. Then each tooth was dried under an air stream and the sealant was applied.

Group 3 (nano-HA 0.03%): The procedures were similar to group 2, except that the solution contained nano-HA at 0.03% concentration.

Group 4 (nano-HA 0.15% + SHMP 0.05%): The solution powder contained nano-HA 0.15% and SHMP 0.05%, which were mixed together before the solvent was added. The other procedures were similar to groups 2 and 3.

Group 5 (nano-HA 0.03% + SHMP 0.01%): The procedures were similar to group 4, except that the solution contained nano-HA at 0.03% and SHMP at 0.01%.

### Microleakage assessment

A total of 60 selected demineralized teeth were randomly divided into 5 groups of 12 teeth each and pretreated as explained above. After the sealant was applied and cured, the teeth were aged in a thermocycling bath at temperatures between 5 °C and 55 °C for 1000 cycles with a dwell time of 30 s and a 15 s transit time between baths. Two samples in each group were randomly selected for SEM evaluation.

Next the root apices were sealed and each tooth surface was covered with two layers of nail polish, leaving a 1-mm margin around the sealant. Then the samples were immersed in 0.5% basic fuchsin dye (Merck, Darmstadt, Germany) solution for 24 h, rinsed and sectioned buccolingually across the sealant area with a diamond saw (Mecatome, Presi, Eybens, France) to obtain two halves of the sealant. Two dentists who were trained prior to the study observed the sectioned teeth under a calibrated digital microscope (Dino Lite, Taipei, Taiwan) at 50× magnification. The proportion of microleakage was calculated by dividing the total distance of dye penetration (in mm) by the total length of the enamel sealant interface (in mm) [27].

### Scanning electron microscopy evaluation

After the sealant was applied on the occlusal surface and the teeth were aged, two samples from each group were randomly selected to evaluate resin infiltration and the interface area. The teeth were sectioned transversally to the sealant–tooth interface and polished with 400, 600, 1000 and 2000 grit silicon carbide paper (SCi) with water cooling. Then the tooth surface was treated with 37% phosphoric acid for 10 s, rinsed for 30 s, and immersed in 5% NaOCl for 2 min. After rinsing, the teeth were dehydrated with ethanols. Next the samples were sputter-coated with gold in a vacuum evaporator, and the interfaces were examined with SEM (VEGA, Tescan, Brno, Czech Republic) at 1500× magnification.

### Shear bond strength test

The root of 100 teeth was sectioned perpendicular to and 2 mm below the cementoenamel junction with a diamond saw. Then the enamel specimens were prepared in blocks so that the crown was embedded in acrylic resin while the buccal surface remained exposed. To obtain a flat surface the exposed enamel was polished with 600–1000 grit waterproof SCi. Each sample was demineralized, and the enamel was sonicated for 10 min to remove any debris due to surface polishing [24]. Then the teeth were randomly divided into 5 groups and pretreatment was applied to each sample as described above. Next a rubber cylindrical mold 3 mm in internal diameter and 3 mm in height was placed on the treated enamel and filled with fissure sealant material, which was then cured. Then the teeth were stored in humid conditions at 37 °C for 24 h, and SBS tests were done with a universal testing machine (Zwick-Roell, Zwic, Ulm, Germany). The load speed applied in a direction parallel to the bonded interface was 1 mm/min until failure occurred, and load failure was recorded in megapascals (MPa). Failure mode of the fractures was evaluated by two previously trained observers under blind conditions with a digital microscope (Dino Lite) at 25× magnification. The types of bond failure were recorded as follows: 1) adhesive fracture at the sealant–enamel interface, 2) cohesive fracture in the substrate (enamel or sealant), 3) mixed fracture when both adhesive and cohesive fractures occurred.

### Energy dispersive X-ray spectroscopy and field emission scanning electron microscopy

A total of 32 teeth were selected and randomly divided into 4 groups of 8 specimens. Each tooth was sectioned transversally in the mesiodistal direction to obtain baseline sound enamel (the enamel surface of the lingually sectioned portion). Enamel blocks were prepared as described above. Then the buccal half of the sectioned teeth was cut in the buccolingual direction to obtain two quarters of the tooth. All parts of each tooth were sonicated for 10 min. The two buccal quarters were demineralized as described above. One quarter of each sample was pretreated (remineralized) as explained above, and the other quarter was used to study demineralization. Next the mineral content of each sample was measured by EDS at baseline (in sound enamel), after demineralization, and after immersion in nano-HA solution (remineralization). The sound sectioned part of each specimen was used as a reference for comparisons with the demineralized and remineralized enamel specimens. In other words all comparisons were done in all 32 tooth specimens.

**Fig. 1:**
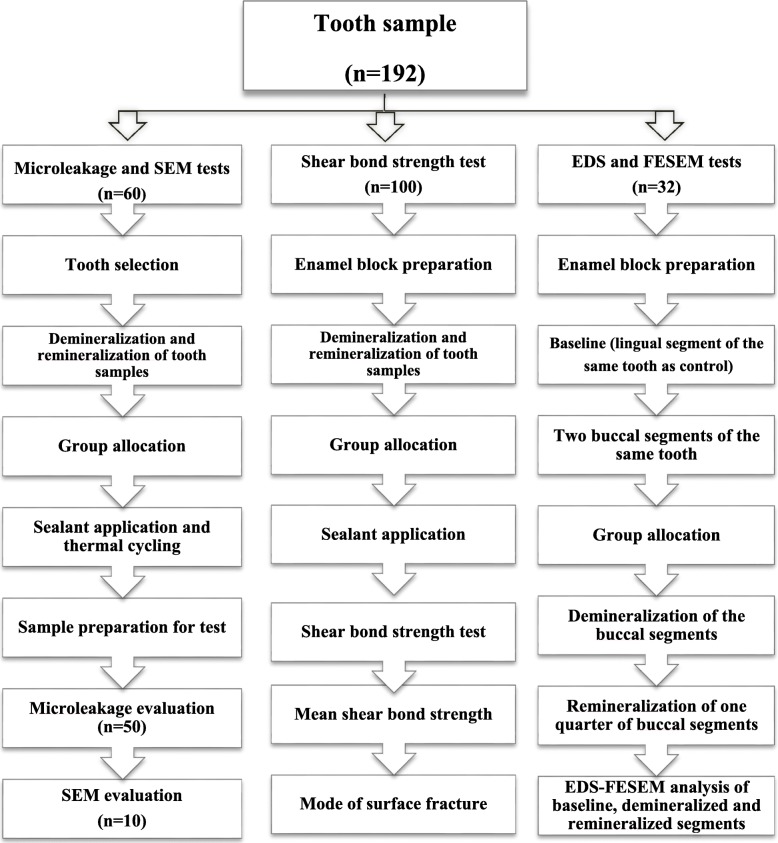
Flowchart showing the methods used in this study

Before EDS analysis, the teeth were carbon coated. The scanning parameter settings and tooth position were held constant to standardize the analytical method and obtain comparable results. The spot size was kept at 100 nm for EDS and 2–3 nm for FESEM. The surface area assessed in each FESEM scan was 1.38 μm (at 75000×) at 15 kV. Scan duration was 45 s. In addition, EDS associated with high-resolution FESEM (MIRA3, Tescan, Brno, Czech Republic) was used for samples in groups 2 to 5 to observe nanoparticles in each sample (Fig. 1).

### Statistical analysis

All data are presented as means ± standard deviations (SD), and were obtained with SPSS version 22.0 (IBM SPSS) software. Microleakage and SBS comparisons were done with the Kruskal–Wallis, one-way analysis of variance (ANOVA) and Duncan’s post hoc tests. Repeated measures ANOVA, the Sidak and Tukey post hoc tests were used to compare the effect of different nano-HA compositions and conditions. *P* values < 0.05 were considered statistically significant.

## Results

### Microleakage and shear bond strength

Table 1 shows the mean (±SD) of the proportion of microleakage values in each group. There were no significant differences between the groups (*P* = 0.939). However, the differences between groups in mean (±SD) SBS were significant (Table 2). Mean SBS in group 2 was significantly greater than in groups 1, 3 and 4 (all *P* < 0.05). There were no significant differences in SBS between group 5 and the other groups. The results of failure mode tests showed that mixed failure was the most frequent mode in all groups.

**Table 1:** Means and standard deviations of the proportion of microleakage in all groups (*n* = 10)

Group	Median (mean ± SD)	Mean rank	*p*-value
1. Etch + FS	0.0 (0.09 ± 0.17)	52.2	0.939
2. Etch + 0.15% nano-HA + FS	0.0 (0.09 ± 0.18)	52.3	
3. Etch + 0.03% nano-HA + FS	0.0 (0.08 ± 0.23)	49.7	
4. Etch + nano-HA 0.15% + SHMP 0.05% + FS	0.0 (0.08 ± 0.23)	50.9	
5. Etch + nano-HA 0.03% + SHMP 0.01% + FS	0.0 (0.05 ± 0.13)	47.3	

Abbreviations: *nano-HA* nanohydroxyapatite, *FS* pit and fissure sealant, *SHMP* sodium hexametaphosphate

**Table 2:** Means and standard deviations of shear bond strength (unit MPa; *n* = 20) after different enamel pretreatments

Group	Mean ± SD	Duncan’s test^a^
1. Etch + FS	14.6 ± 2.5	A
2. Etch + 0.15% nano-HA + FS	16.8 ± 2.7	B
3. Etch + 0.03% nano-HA + FS	14.2 ± 2.1	A
4. Etch + nano-HA 0.15% + SHMP 0.05% + FS	14.0 ± 2.2	A
5. Etch + nano-HA 0.03% + SHMP 0.01% + FS	15.1 ± 2.5	AB
*p*-value	0.010	–

^a^Mean values with different capital letters were significantly different

Abbreviations: *nano-HA* nanohydroxyapatite, *FS* pit and fissure sealant, *SHMP* sodium hexametaphosphate

### Sealant penetration

**Fig. 2:**
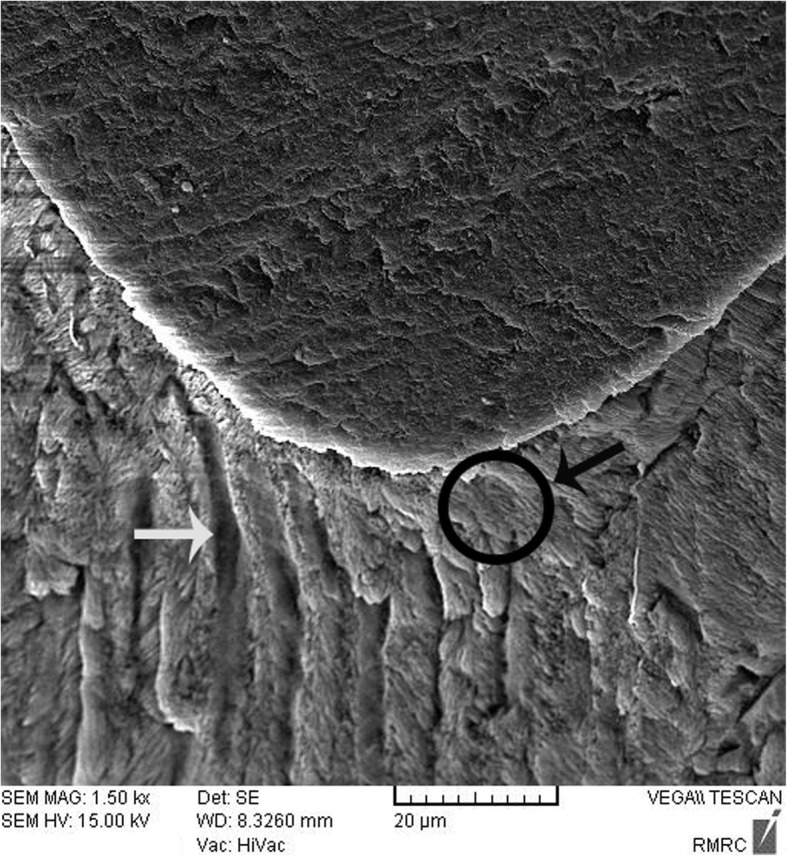
SEM images of demineralized etched enamel and resin sealant adaptation on the interface (group 1). The white arrow shows the non-infiltrated area. The black arrow and circle show the infiltrated area. Abbreviations: SEM, scanning electron microscopy

**Fig. 3:**
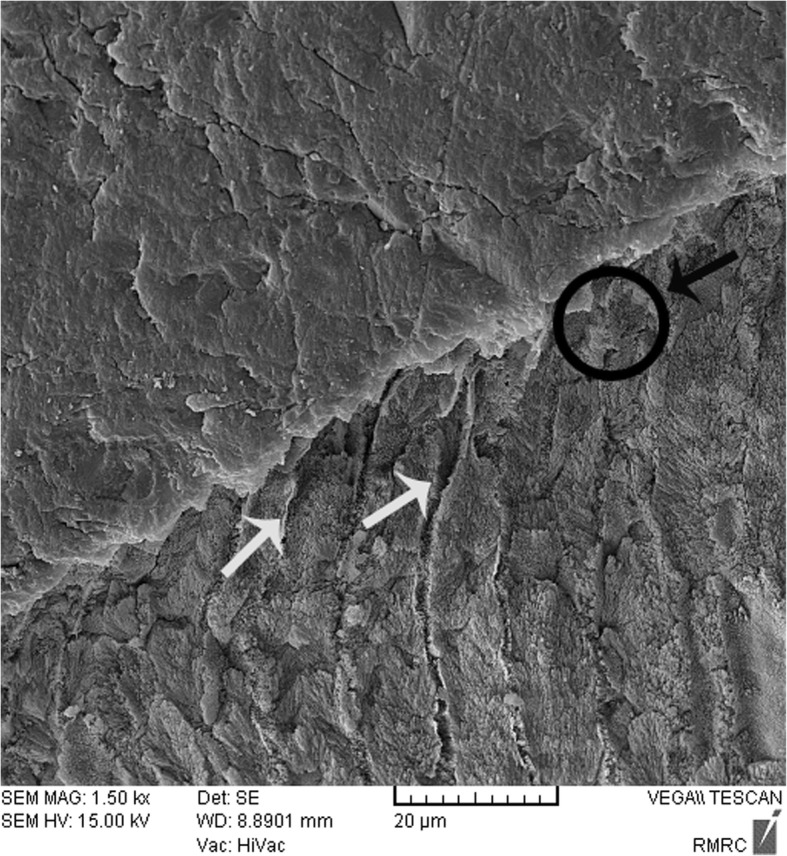
SEM image of nano-HA (0.15%) deposition on the pretreated enamel surface (group 2). The white arrow shows the non-infiltrated area. The black arrow and circle show the infiltrated area. Abbreviations: nano-HA, nanohydroxyapatite

**Fig. 4:**
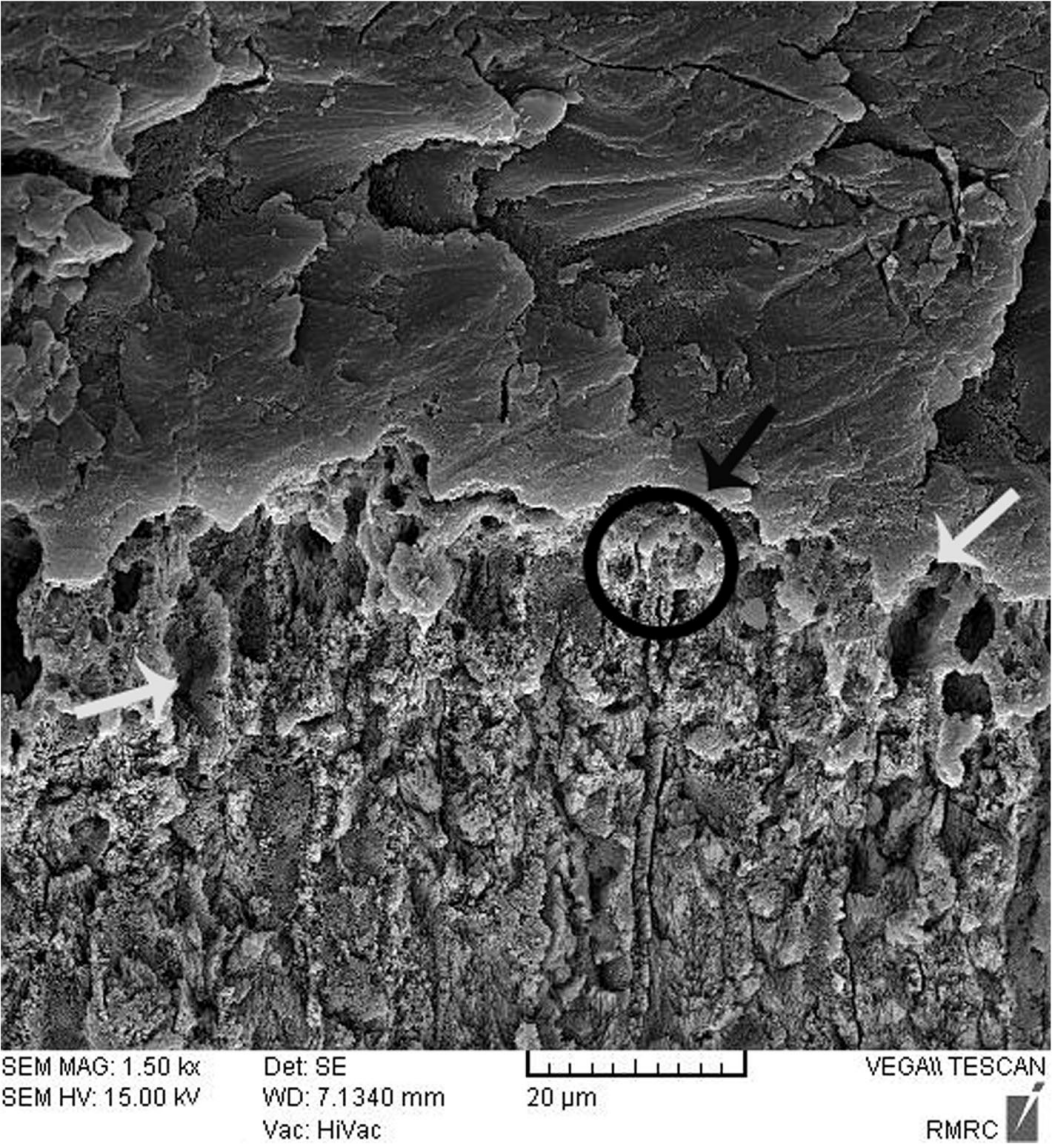
SEM image of nano-HA (0.03%) deposition on the tooth surface, showing absence of nanoparticle infiltration in some areas (group 3). The white arrow shows the non-infiltrated area. The black arrow and circle show the infiltrated area

**Fig. 5:**
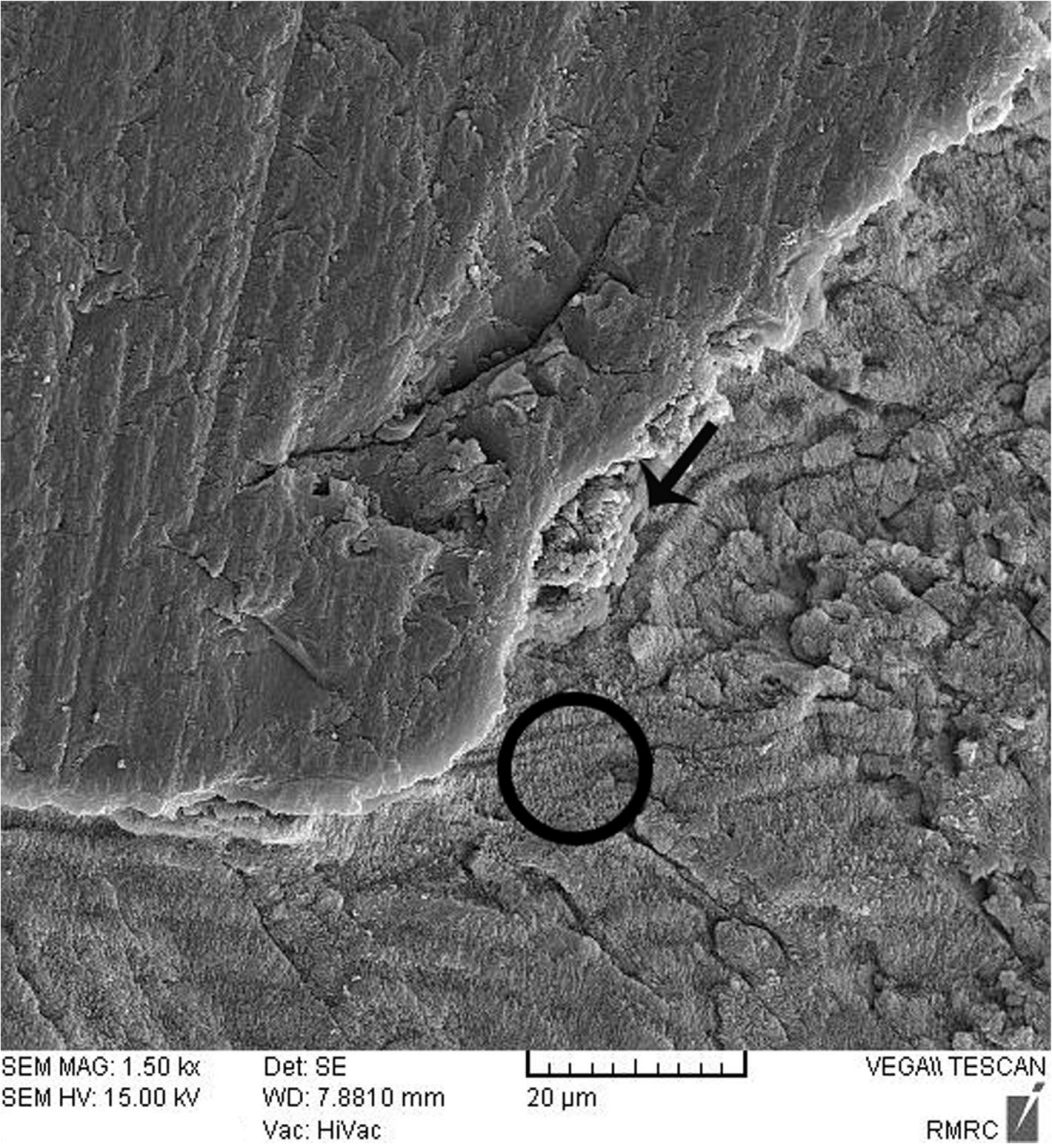
SEM image of nano-HA dispersal across a larger area in group 4 (nano-HA 0.15% + SHMP 0.05%) compared to group 2. The black arrow and circle show the infiltrated area

**Fig. 6:**
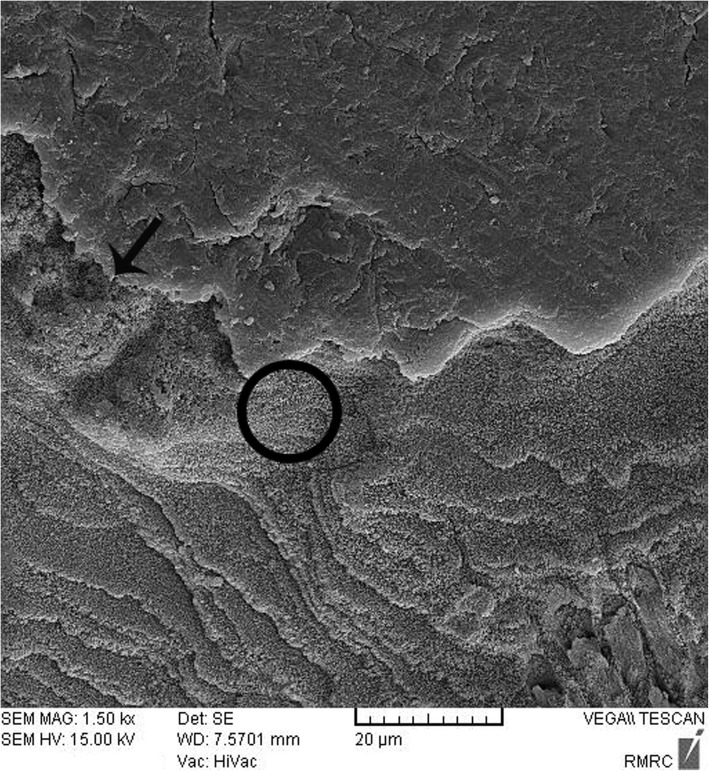
SEM image of nano-HA dispersal across a larger area in group 5(nano-HA 0.03% + SHMP 0.01%) compared to group 4

Sealant penetration was investigated with SEM in the deepest part of the pit and fissures in group 1. The prism-like appearance well was defined due to the demineralization process and effect of etching. The spaces were filled with resin sealant (Fig. 2). Applying nano-HA 0.15% created a layer that covered the surface and penetrated into parts of the etched enamel (Fig. 3). The use of nano-HA 0.03% led to resin infiltration in parts of the fissures, although some parts showed no penetration (Fig. 4). Applying SHMP in group 4 (Fig. 5) and group 5 (Fig. 6) led to increased dispersal of the nanoparticles over a larger area, with no increase in the depth of infiltration in the fissures.

### Mineral content

**Fig. 7:**
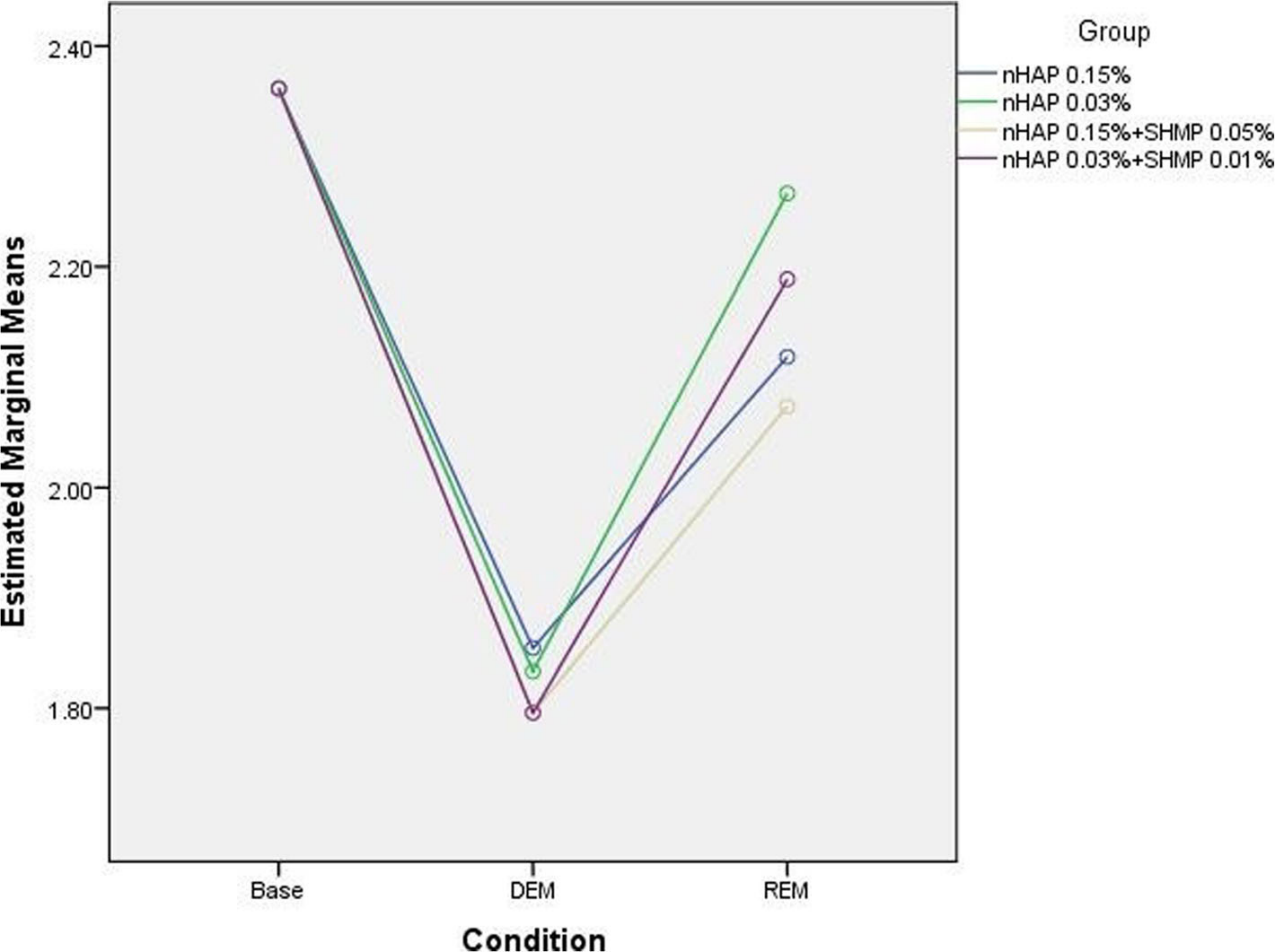
Mean Ca/P wt% ratio in experimental groups under different conditions

Mineral contents in each sample at all three steps were measured by EDS. There was no significant interaction between compositions and conditions (*P* = 0.794). In other words, the effect of different conditions on the Calcium (Ca)/Phosphate (P) ratio did not depend on the type of composition used (Fig. 7). There were significant differences in Ca/P weight percentage (Wt%) ratio between baseline, demineralized and remineralized enamel (*P* < 0.001) (Table 3). The highest ratio was recorded for sound enamel and the lowest for demineralized enamel. There were no significant differences in Ca/P Wt% ratio between groups 2, 3, 4 and 5 regardless of the conditions (*P* = 0.582).

**Table 3:** Means and standard deviations of Ca/P Wt/% ratio in groups 2 to 5

	Condition				
Group	Baseline	DEM	REM	Total	*p*-value
2	2.4 ± 0.1	1.8 ± 0.2	2.1 ± 0.3	2.1 ± 0.1	0.582
3	2.4 ± 0.1	1.8 ± 0.1	2.3 ± 0.3	2.1 ± 0.1	
4	2.4 ± 0.0	1.8 ± 0.3	2.1 ± 0.4	2.1 ± 0.2	
5	2.4 ± 0.1	1.8 ± 0.1	2.2 ± 0.2	2.1 ± 0.1	
Total	2.4 ± 0.1	1.8 ± 0.2	2.2 ± 0.3	*p*-value< 0.001	

Abbreviations: *wt%* weight percentage, *DEM* Demineralization, *REM* Remineralization

**Fig. 8:**
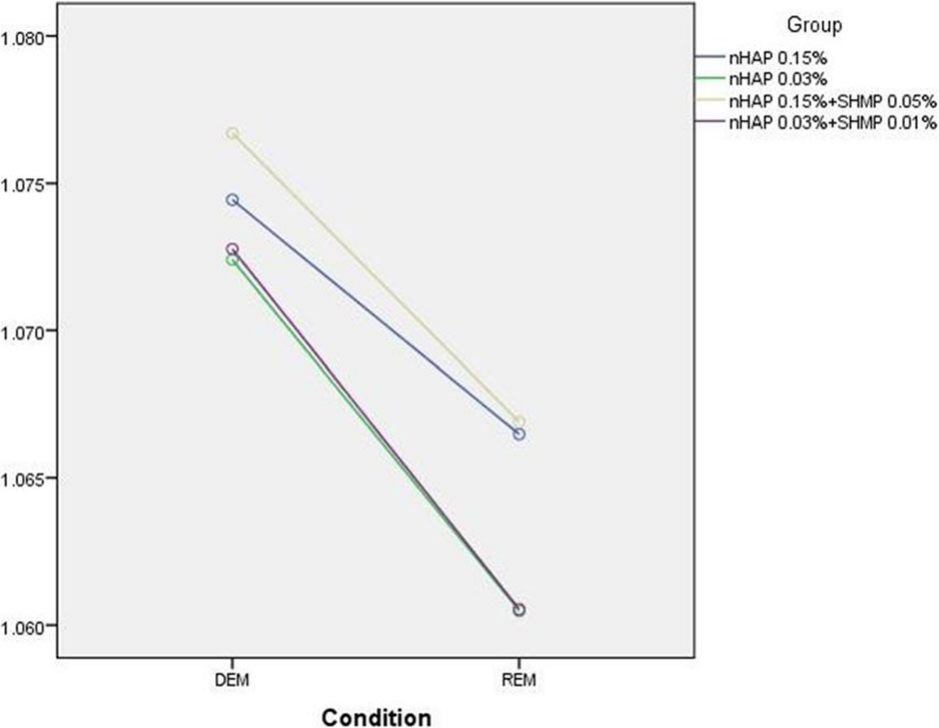
Mean ZAF-corrected Ca/P ratio in experimental groups under different conditions

The standardless EDS value with ZAF correction for matrix effects (EDS-ZAF) was also recorded. There was no significant interaction between compositions and conditions (*P* = 0.385). In other words, the effect of changing ZAF for Ca/P from demineralization to remineralization did not depend on the type of composition used (Fig. 8). The results showed significant differences in ZAF Ca/P ratios between all groups (*P* < 0.001) and between groups 2 to 5 (*P* = 0.004). There were significant differences in ZAF-corrected Ca/P ratios between groups 4 and 3 (*P* = 0.014) and groups 4 and 5 (*P* = 0.019). However, there was no significant difference between groups 4 and 2 (*P* = 0.857). Also regardless of the group, the ratio in remineralized enamel was significantly higher compared to after demineralization (*P* < 0.001). Table 4 shows the mean ZAF Ca/P ratios in different conditions and groups.

**Table 4:** Means and standard deviations of ZAF-corrected Ca/P ratios in groups 2 to 5

	Condition			
Group	DEM	REM	Total	*p*-value
2	1.074 ± 0.006	1.072 ± 0.007	1.070 ± 0.005 AB	0.004
3	1.077 ± 0.006	1.073 ± 0.007	1.066 ± 0.004 A	
4	1.074 ± 0.006	1.066 ± 0.005	1.072 ± 0.004 B	
5	1.060 ± 0.003	1.067 ± 0.006	1.067 ± 0.003 A	
Total	1.074 ± 0.006	1.064 ± 0.005	p-value< 0.001	

Abbreviations: *DEM* Demineralization, *REM* Remineralization

### Nanoparticle micromorphology

**Fig. 9:**
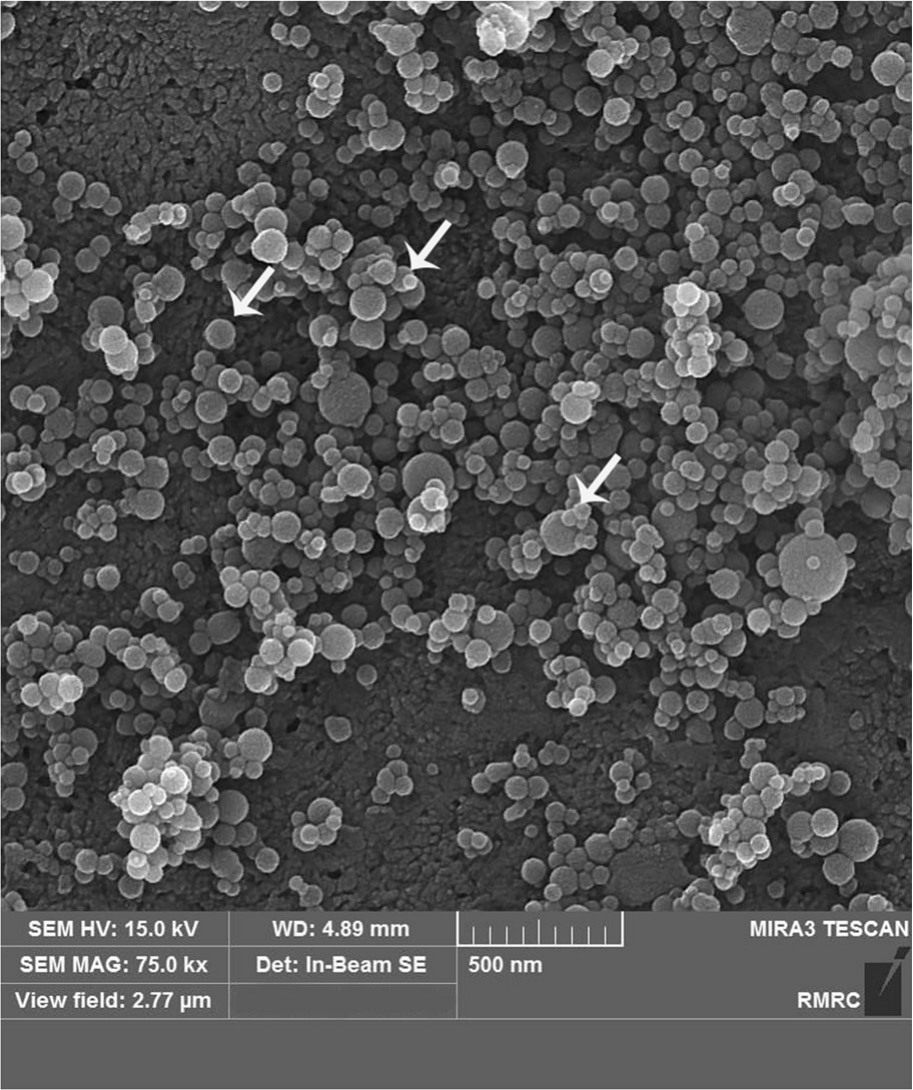
Field emission scanning electron microscopic (FESEM) image from group 2 showing spherical nanoparticles dispersed on the enamel. The white arrow shows spherical nanoparticles

**Fig. 10:**
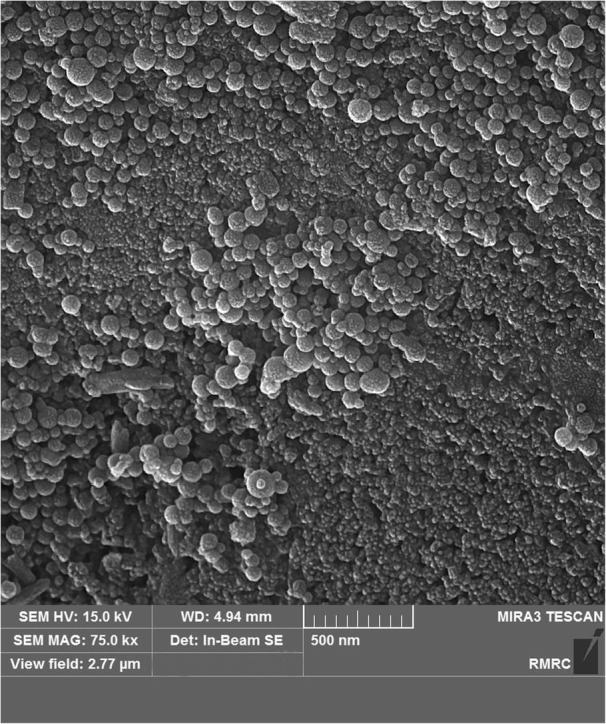
FESEM image from group 3 showing fewer nanoparticles and less particle clustering

**Fig. 11:**
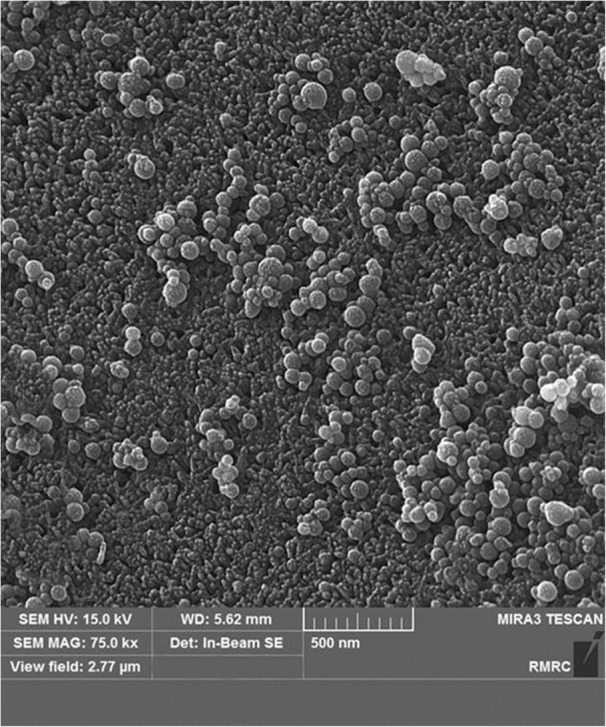
FESEM image from group 4 showing spherical nanoparticles dispersed on the enamel

**Fig. 12:**
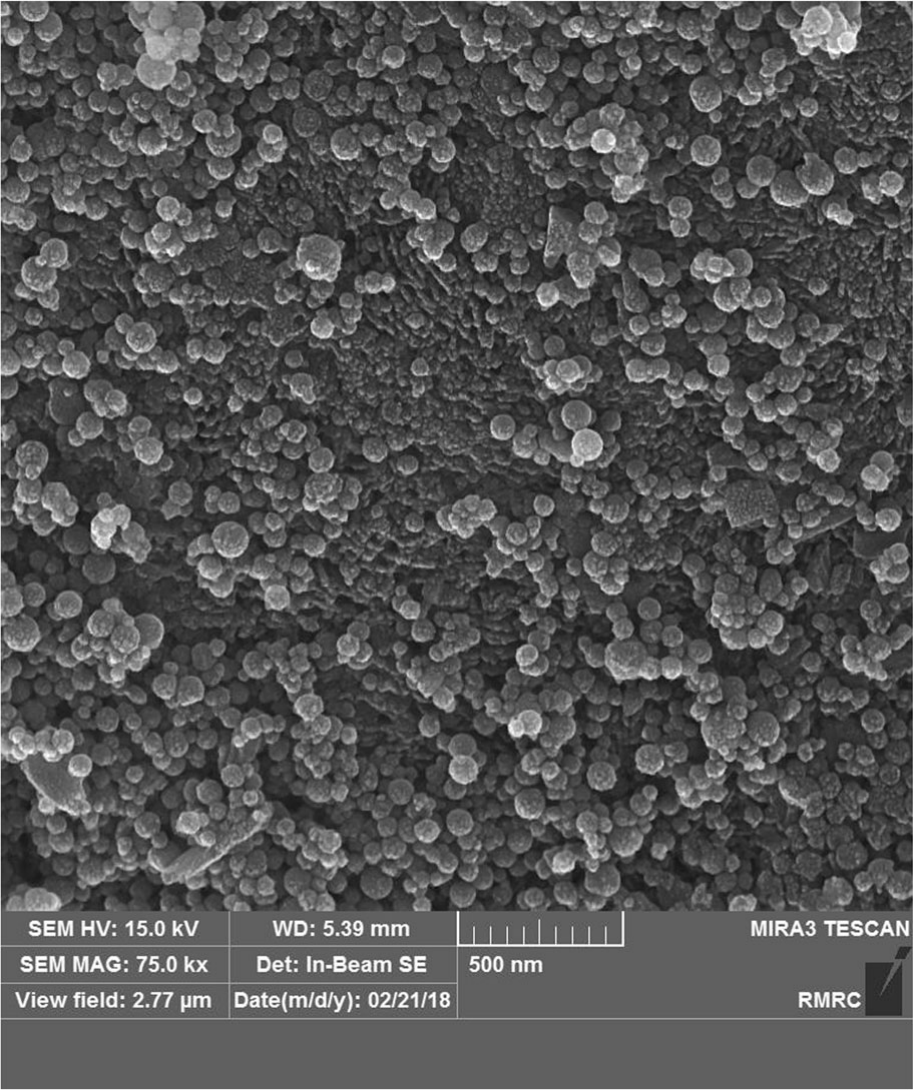
FESEM image from group 5 showing nano-HA dispersal without cluster formation

Nano-HA particles were spherical in all the interventional groups. The structure was porous, and large numbers of nanoparticles were dispersed on the surface in group 2 (Fig. 9). The FESEM images for group 3 revealed a similar appearance except with fewer nanoparticles and less particle clustering (Fig. 10). Applying SHMP (groups 4 and 5) resulted in nano-HA dispersal over a larger surface area and less clustering (Figs. 11 and 12) compared to the groups without SHMP.

## Discussion

The present results showed no significant difference between intervention groups and the control group (FS alone), which was in accordance with our null hypothesis. The higher concentration of nano-HA (0.15%) led to greater SBS compared to the control group; however, there was no significant difference between the control group and the group with the lower concentration (0.03%) of nano-HA. Thus the null hypothesis (H0) was partially rejected.

Guidelines have recommended using a pit and fissure sealant on sound intact enamel and noncavitated (incipient caries) lesions [2, 3]. Some materials, such as nanoparticles, have been found to increase enamel remineralization in sealant therapy [13–16].

Nano-HA [Ca^10^(PO_4_)^6^(OH)^2^] is similar in structure to the main mineral component of teeth and bone, and products containing nanoparticles have led to enhanced precipitation of calcium and phosphate ions in the tooth structure [14, 20, 28]. Some studies reported that a suspension containing 10% nano-HA particles (10–20 nm diameter) enhanced remineralization of the superficial layer in initial caries lesions to a depth of 20–40 μm. However, little remineralization was seen in the body of the lesion [29, 30]. One earlier study found that nano-HA (20 nm size and dimension up to 100–150 mm) enhanced remineralization in the subsurface of initial lesions, particularly in dentin compared to enamel [23]. In the present study the mean diameter of nano-HA was 10.67 nm.

Factors that influence the success of sealant treatment include marginal adaptation between the tooth and fissure sealant, and bond strength of the materials [9, 27]. In the present study all groups showed some microleakage, which is consistent with previous work that found no sealant materials were able to completely eliminate microleakage between the tooth and sealant [30]. Several factors influence microleakage along the sealant and enamel surface, e.g. pit and fissure morphology, sealant viscosity and method of FS application, all of which impact sealant infiltration [31]. Other factors include the enamel pretreatment technique [6, 7], intensity of light curing [31], and mechanical properties of the sealant materials [31, 32]. Resin polymerization shrinkage as well as thermal dimensional changes in fissure sealants [33] may also lead to microleakage and gap formation. We observed no significant differences between the interventional groups (groups 2 to 5) and the control group without nano-HA application. This result is consistent with a previous study in which the use of products containing acetone prior to sealant application led to similar results for sealant adaption compared to conventional acid etching [30]. These findings may be due to the presences of acetone as a solvent in the nano-HA solution. Acetone is a colorless liquid that evaporates rapidly and can be mixed with most organic solvents and with water. Nano- HA solutions containing acetone and water, therefore, may improve resin infiltration into the demineralized enamel [24]. Another explanation for these results may be the lack of a significant effect of nanoparticles on the sealing capacity of the sealant.

In the present study SBS differed significantly between group 2 (nano-HA) and groups 1 (control), 3 and 4. This may reflect the ability of the hydrophobic resin sealant to enhance surface wettability of the etched enamel as a result of nano-HA properties [16] and the presence of acetone in the solution. Hydroxyapatite “acts as a drug delivery carrier due to superior adsorptive properties”, and this may in turn increase bond strength [14]. Another explanation for the increased bond strength may be the creation of a reactive layer between the resin sealant and nano-HA. In group 2 the higher concentration of nanoparticles (0.15%) may have contributed to the greater SBS. In agreement with our study, Borges et al. showed that applying casein phosphopeptide-amorphous calcium phosphate (CPP-ACP) products beneath the fissure sealant promoted bond strength between the resin sealant and the enamel [12].

After enamel demineralization, the loss of minerals such as calcium and phosphorus results in voids or gaps on the tooth surface. The acid etch technique also creates microporosities on the enamel surface. Both processes provide spaces that facilitate resin infiltration and suitable bonding between the resin and tooth. When nano-HA solution was used, the ionic form of Ca and P enters the porous enamel and provides seeds for remineralization [25, 28, 34, 35]. SEM images in the present study showed differences between groups in the degree of penetrations of the resin sealant into these spaces.

Energy dispersive X-ray spectroscopy is a chemical elemental microanalysis technique to measure mineral content at the ultrastructural level, and has been used to investigate the remineralization capacity of different materials [14, 17, 24]. In the present study we used FESEM instead of SEM to obtain more detailed structural data of higher quality and resolution [35]. Our analysis of EDS data focused on changes in Wt% of Ca, P and the Ca/P ratio, given that these ions are important indicators of the effect of nano-HA on enamel remineralization. To enhance the accuracy of our EDS analysis, we compared three portions of each tooth sample at each of three steps: baseline (sound enamel, as a reference), after demineralization, and after remineralization. In addition we obtained the ZAF-corrected Ca/P ratio to further increase the accuracy of the EDS results.

Our EDS results showed that nano-HA released Ca and P ions onto the demineralized surface, in accordance with previous studies [18, 24]. The lowest Ca/P Wt% ratios were seen (not unexpectedly) in demineralized enamel. There were no significant differences in this ratio between groups – a finding consistent with earlier EDS results for Ca/P ratio in a study of nano-HA in demineralized dentin [24]. This may explain, in part, our finding that Wt% Ca/P did not differ among our experimental groups. Although SHMP and acetone increase solubility of nano-HA and would thus be expected to enhance nanoparticle infiltration [24], SHMP did not increase nanoparticle infiltration in deeper parts of the enamel in the present study.

In the present study the ZAF-corrected Ca/P ratio in demineralized enamel was higher than in the interventional groups, as a foreseeable result of the demineralization process. Among the interventional groups, the ZAF-corrected ratio in group 4 was significantly higher than in groups 3 and 5. This may be related to the higher values of P (i.e. the higher concentration of SHMP) in group 4 (0.15% nano-HA + 0.05% SHMP). In dentistry, SHMP is used as a deflocculant to limit nanoparticle agglomeration or reduce the size of nano-HA agglomerates to less than 5 nm [24]. Our SEM observations showed that in groups 4 and 5 (both with SHMP) the nanoparticles were better dispersed on the surface of the sealant–enamel interface than in groups 2 and 3 (without SHMP). In group 1 (control without nano-HA) the sealant penetrated to the deepest parts of pits and fissures. When the higher concentration of nanoparticle was used (group 2), a layer of nano-HA was seen on the treated enamel – a finding consistent with an earlier study with tricalcium phosphate nanoparticles [15]. Group 4 also used nano-HA at 0.15%, together with SHMP, and this combination may have influenced our SBS and SEM results. However, the lack of earlier studies centered on nanoparticles in treated enamel precludes further comparisons with previous findings.

Nanoparticle micromorphology was investigated in FESEM images, which showed that they were spherical as reported in a previous study [18]. In group 2 (with the higher concentration of nano-HA), FESEM also showed larger amounts of nanoparticles dispersed on the surface area. In groups 4 and 5 (with SHMP), FESEM images showed more nanoparticles which were well dispersed on a larger surface area compared to groups 2 and 3. Earlier studies found that nonagglomerated particles are better able to penetrate into the surface and prevent nanoparticle precipitation [17, 25]. However, nanoparticle penetration into the deeper parts of the tooth was insufficient, as also reported by others [15].

A potential limitation of this study was that laboratory tests may not accurately reflect clinical conditions. To reduce the influence of confounding factors, we used thermocycling, which replicates actual clinical procedures. Another limitation is the lack of studies designed to investigate the effects of nanoparticles used prior to sealant application; this precluded comparisons between our findings and those of other studies. A further potential limitation is the approach we used to prepare the materials tested in each group. Therefore a pilot analysis was done to determine the appropriate concentrations of nano-HA and SHMP for testing before the experimental specimens were used for data collection and analysis. Our pilot study was based essentially on the methods of Besinis et al., and we implemented their 3:1 ratio of nano-HA to SHMP [24, 25]. However, we tested different concentration of nano-HA to obtain more complete results. The results of the present study support our hypothesis that nano-HA would be able to remineralize enamel and would enhance SBS without influencing the sealant’s sealing ability. Finally, we recommend additional in vitro and in vivo studies to determine the advantages and drawbacks of applying nano-HA before the fissure sealant in terms of clinical outcomes.

## Conclusions

The use of nano-HA before fissure sealant application may be an effective method to fill porous spaces in demineralized enamel pits and fissures, and to enhance remineralization compared to demineralized enamel. Moreover, the higher concentration of nano-HA (0.15%) led to increased shear bond strength of the sealant compared to conventional acid etching and sealant application. Both concentrations of nano-HA (0.15 and 0.03%) with or without SMHP led to enhanced remineralization compared to demineralized enamel.

## Abbreviations

Ca: Calcium
CPP-ACP: Casein phosphopeptide-amorphous calcium phosphate
EDS: Energy-dispersive X-ray spectroscopy
FESEM: Field emission scanning electron microscope
FS: Pit and fissure sealant
Nano-HA: Nanohydroxyapatite
P: Phosphate
SBS: Shear bond strength
SCi: Silicon carbide paper
SEM: Scanning electron microscopy
SHMP: Sodium hexametaphosphate
